# The electrocardiographic abnormalities in highly trained athletes compared to the genetic study related to causes of unexpected sudden cardiac death

**Published:** 2009-11-25

**Authors:** C Macarie, I Stoian, D Dermengiu, L Barbarii, I Tepes Piser, O Chioncel, A Carp, I Stoian

**Affiliations:** * ‘Prof. Dr. C.C. Iliescu’ National Institute of Cardiovascular Diseases, BucharestRomania; ** ‘Mina Minovici’ National Institute of Forensic Medicine, BucharestRomania; *** National Institute of Sports Medicine, Bucharest Romania

**Keywords:** endurance athlete, electrocardiography, genetic analysis

## Abstract

**Background**: Electrocardiograms in elite endurance athletes sometimes show bizarre patterns suggestive of inherited channelopathies (Brugada syndrome, long QTc, catecholaminergic polymorphic ventricular tachycardia) and cardiomyopathies (arrhythmogenic right ventricular cardiomyopathy, hypertrophic cardiomyopathy) responsible for unexpected sudden cardiac death. Among other methods, genetic analyses are required for correct diagnosis.

**Objective**: To correlate 12– lead electrocardiographic patterns suggestive of inherited channelopathies and cardiomyopathies to specific genetic analyses.

**Design**: Prospective study (2004–2007) of screening 12–lead ECG tracings in standard position and higher intercostal spaces V1 to V3 precordial leads, performed in athletes and normal sedentary subjects aged match. Genetic analyses of subjects with ECG abnormalities suggested inherited channelopathies and cardiomyopathies.

**Setting**: All cardiologic exams and electrocardiograms were performed at ‘Prof. Dr. C.C. Iliescu’ National Institute of Cardiovascular Diseases (Bucharest, Romania). The genetic studies were done at ‘Mina Minovici’ National Institute of Forensic Medicine (Bucharest, Romania).

**Participants**: 347 elite endurance athletes (seniors–190, juniors–157), mean age of 20; 200 subjects mean age of 21, belonging to the control group of 505 normal sedentary population.

**Results**: Seniors. RSR' (V1 to V3) pattern, in 45 cases (23.68%), 5 of them with questionable Brugada sign (elevated J wave and ‘coved’ ST segment,< 2mm in one lead, V1. Typically, Brugada 1 sign was found in one case (0.52%) with no SCN5A abnormalities. One athlete (0.52%) had normal ECG and exon1 SCN5A duplication. MRI confirmed three arrhythmic right ventricular cardiomypathy epsilon waves (1.57%), in one case. ST–segment elevation myocardial injury like in V1–V3 precordial leads in 34 athletes (17.89%).Genetic analyses–no gene mutations.

**Juniors** Upright J wave was found in 43 cases (27.38%). Convex ST segment elevation in V1–V3/V4, in 39 cases (24.84%). Bifid T wave with two distinct peaks was found in 39 cases (24.84%), 5 of them with mild prolonged QTc (0.48 ‘–0.56’) and KCN genes mutations. Nine (5.73%) of the elevated ST segment juniors had questionable Brugada sign, two of which with KCN (n=1) and SCN5A (n=1) gene mutations. Ajmaline provocative test was negative in 4 and was refused by 5 subjects.

**Conclusion**: Bizarre QRS, ST–T patterns suggestive of abnormal impulse conduction in the right ventricle, including the right outflow tract, associated with prolonged QTc interval in some cases were observed in highly trained endurance athletes. The genetic analyses, negative in most athletes, identified surprising mutations in SCN5A and KCN genes in some cases.

The medical society and athletes' community are extremely interested in the precocious identification of athletes' risk of sudden cardiac death. Pre– participating screening guidelines for athletic training are formulated by many scientific medical groups [1–5, 7– 10]. The frequent causes of sudden cardiac death in athletes younger than 35 years old are hypertrophic cardiomyopathy (HCM) 50%, idiopathic left ventricular cardiomyopathy 18%, coronary artery abnormalities 14%, arrhythmogenic right ventricular cardiomyopathy (ARVC), 2.23–20% [11, 57]. In 3%, sudden cardiac death is associated with normal structural hearts. Inherited cardiac channelopathies (Brugada syndrome, long QT syndroms, idiopathic ventricular fibrillation, polymorphic ventricular tachycardia) could be the causes for sudden cardiac death [11– 14]. In the absence of any echocardiographic structural abnormalities, establishing the diagnosis is often difficult to achieve when the electrocardiograms of trained athletes often present findings considered abnormal by usual standards. The correct management includes detailed athlete's history, 12–lead electrocardiography, additional tests (i.e. 24h heart rhythm ambulatory monitoring, Ajmaline provocative test) and sometimes, targeted genetic analysis [15– 18]. A genotype – phenotype analysis based on the electrocardiographic findings in elite endurance athletes was the purpose of our study.

## Material and methods

### Studied population

Endurance athletes: 347 intensively trained athletes (Caucasians) participants in sports activity with high cardiovascular burden were chosen [19, 20]. The sports were canoeing, rowing, football, hockey, tennis, swimming, and athletics. There were 190 senior and 157 junior athletes [20]. Senior athletes had trained intensively for 20–27 h /week for>5 years and participated in World Championships and Olympic Games. The junior primary and final selections were at the age of 12–14 years old and 17–18 years old respectively.

Sedentary healthy population (controls): The apparently normal (asymptomatic, normal physical examination, no detectable cardiovascular risk factors) sedentary population [21, 25] of 505 subjects and participants were investigated for work eligibility in our Cardiology Department (Auto/Work License). Exclusion criteria for control subjects were coronary artery disease, valvular / congenital diseases, cardiomyopathies, heart failure, and cardiovascular drug therapy. None of the athletes and controls received any drugs. The protocol was approved by the Hospital Ethical Committee and informed consents were obtained from all subjects who enrolled in the study.

### Study protocol

Clinical examination: The cardiologic examination included detailed personal and family history and complete physical examination [9,11].

Standard 12–lead electrocardiography: ECG was recorded with a digital machine (MACC 5500 GE Medical Systems, Milwaukee, Wisconsin, USA) in all athletes and controls. ECG measurements were computer–assisted and independently manually controlled by two experienced electrocardiographic readers (CM,IS) unaware of clinical data and sports categories [22–24,36]. Disagreements regarding the measurements were resolved by consensus. An average of 3–5 cardiac cycles was used. Tracings were obtained readers (CM,IS) unaware of clinical data and sports categories [22–24,36]. Disagreements regarding the measurements were resolved by consensus. An average of 3–5 cardiac cycles was used. Tracings were obtained > 24 hours after the last athletic activity. Recordings in higher right precordial V1–V3 intercostal spaces (six new positions:–1V1 to–1V3;–2V1 to–2V3) to detect the Brugada signs were performed in all athletes and sedentary normal subjects [26, 27,32]. Ajmaline provocative test in standard 12–lead ECG and upper right V1–V3 precordial leads positions was indicated according to the current guidelines [33,61]. ECG parameters were evaluated according to actual criteria [22–24,36].

### Standard electrocardiographic variables. Definitions

We used the current electrocardiographic criteria [22,23] including:

1. normal sinus rhythm: heart rate 60–100 bpm;
2. normal QTc (Bazett) < 0.39‘in men ,<0.44‘ in women;
3. borderline normal ECG : RSR' or rSr' pattern in V1 lead (QRS duration < 0.10‘, height < 7mm; r'wave < 2mm and r' < r; juvenile T wave (inversed T in V1–V3 precordial leads; usually deep < 2mm);
4. Q wave (< 0.5 mm in lead Ⅲ; normal duration< 0.03‘ and height< 0.4 mm in all leads);
5. J wave (Osborn; deflection that distort the QRS–ST junction usually in Ⅱ,Ⅲ,V4 to V6 leads);
6. upright T wave (beyond normal< 0.5 mm in limb leads and< 0.10 mm in precordial leads);
7. bifid T wave (T wave with two peaks different from U wave; possible delayed right ventricular repolarization);
8. ST–segment, normally isoelectric (within normal limits: elevation and depression<0.1mm in limb leads, elevation<0.3 mm in V1–V3 precordial leads; measurements at 80 ms from the J point).

### ECG patterns in highly trained endurance athletes [41–45,47–49,54]

1. Distinctly abnormal ECG, strongly suggesting cardiovascular disease:
  1. striking increase in R or S wave voltage (>35mm) in any lead;
  2. Q waves> 4 mm in depth in>2 leads;
  3. inverted T wave>2mm in >2 leads;
  4. left bundle branch block
  5. marked left (>–30 degrees) or right (>110degrees) QRS axis deviation;
  6. Wolff–Parkinson–White pattern.
2. Mild abnormal ECG
  1. increased R or S wave voltage (30 to 34mm) in any lead;
  2. Q waves 2 to 3 mm in depth, in>2 leads;
  3. T wave flat, minimally inverted or particularly tall (i.e.>15mm) in>2 leads;
  4. abnormal R wave progression in the anterior precordial leads;
  5. left atria enlargement;
  6. short PR interval (<0.12‘).
3. Normal ECG or ECG with minor alterations (athlete's heart syndrome):
  1. PR interval duration>0.20';
  2. R or S voltage, 25 to 29mm;
  3. early repolarization;
  4. RSR' pattern in V1 ,V2 of 0.12‘ in duration);
  5. sinus bradycardia < 60 bpm

**For the Brugada syndrome**:long QT syndrome, polymorphic ventricular tachycardia, ARVC and HCM the commonly adopted clinical and electrocardiographic criteria were used in the study [30,31,34,35,37–40,47–49].

**Holter monitoring**, indicated when necessary [28,29,46].

Echocardiography: Echocardiographic, standard Doppler and TDI analyses were performed by digital ultrasound machine (VIVID 3, GE Medical Systems, Milwaukee, Wisconsin, USA; Aloka and Grosound, Japan). Measurements were done according to ASE criteria. [58]

Genetic study: Subjects with positive or questionable ECG patterns of inherited channelopaties / cardiomyopaties were enrolled.

Techniques. Genomic DNA (200 micro l blood)– QiAampDNA blood minikit (Qiagen).

SCN5A and KCN genetic mutations studies were performed with Multiplex Ligation–Dependent Probe Amplification (MLPA, MRC Holland). SALSA P108 for SCN5A and SALSA P114 for KCNQ1, KCNH2, KCNE1, KCNE2 , kit were used. [50,51, 52,53, 54,55, 56]

Statistics: Results are expressed in mean value± SD. Proportions were compared with the chi square test, where appropriate.

## Results

**Clinical characteristics of studied population**

**Fig 1:**
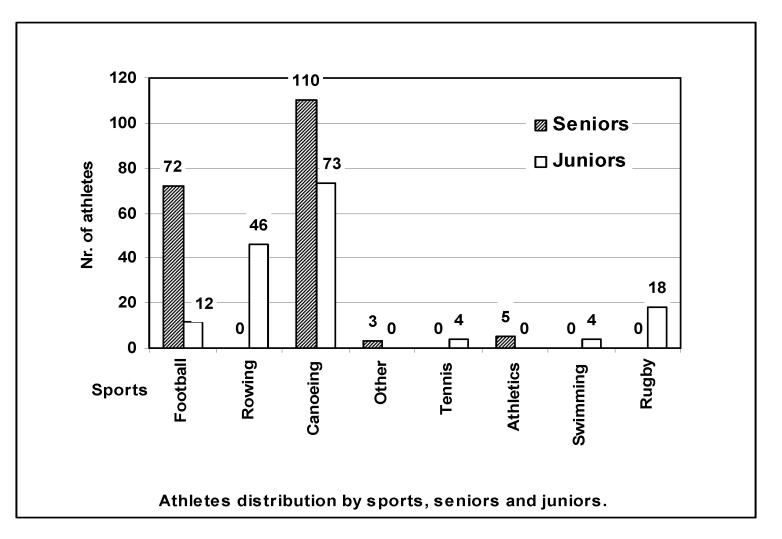
190 seniors and 157 juniors athletes and their distribution according to type of sport

We examined 347 athletes (seniors 190; juniors 157). Fig 1shows the athletes' distribution according to the type of sport and level of training. 

**Athletes**There were 272 males (78.38%) and 75 females (21.6%). The athletes group characteristics: age 19.45 ± 4.82 years old (11–37), height (cm) 178.08 ± 8.99(140–200), weight (kg) 73.70 ± 12.33 (34–109), BSA (m^2^) 1.90 ± 0.19(1.15 – 2.37), blood pressure (mmHg) (110/65–140/85), heart rate (bpm) (32–110). 

**Controls** 505 normal sedentary subjects, 284 males (56.23%), 221 females (43.75%). Characteristics: age 34.93±10.88 years old (13–76), height (cm) 170.71±8.46 (134–195), weight (kg) 68.86±12.25 (29–125), BSA (m^2^) 1.79±0.18 (1.14 – 2.41), blood pressure (mmHg)(105/60–143/85), heart rate (bpm)(42–115). 

The demographic data of the entire cohort of sedentary subjects is summarized in Table 1, according to the decades of age.

**Table 1:** Sedentary normal subjects group divided by decades of age (No., number, BSA, body surface area, BP, blood pressure,systolic/diastolic; HR, heart rate)

Age (y)	No. (505)	Height (cm)	Weight (Kg)	BSA (m^2^)	BP(mmHg) mean	HR(bpm)
13–20	21	163.1±12.61	54.5±11.95	1.58±0.23	105/75	84±12
21–30	179	171.27±11.31	67.33±15.05	1.78±0.19	110/75	82±14
31–40	189	171.37±7.53	70.54±14.43	1.83±0.17	120/80	78 ± 11
41–50	67	168.66±8.36	70.07±10.14	1.79±±0.16	125/75	68 ±15
51–60	35	168.78±8.91	74.12±8.84	1.78±0.35	130/75	72 ±15
61–76	14	172±8.57	77.63±10.34	1.90±0.14	130/85	68±10

### Electrocardiographic findings

All electrocardiographic parameters in athletes (n=347) are summarized in Table 2.

**Senior athletes.** RSR' pattern in high V1–V3 precordial leads in 45 athletes (23.68%) associated with ST elevation in 34 cases (17.89%). Coved ST segment elevation, suggestive for Brugada 1 sign in one lead only (standard/high V1; questionable Brugada sign) in 5 of ST elevation subjects. Provocative Ajmaline test – negative in 2 cases and refused by 3 subjects. Complex premature ventricular beats (Holter, 24h), were found in 23 athletes (12.10%).

Brugada sign1, V1–V3 standard / high, one (0.52%) subject; EP induced ventricular tachycardia.

Epsilon wave, was found in 3 athletes (1.57%). Bifid T wave associated to prolonged QTc (0.48‘ ± 0.09’) was found in 8 athletes (4.21%).

There was a normal echocardiographic exam except for one epsilon subject and for ARVC echo data, diagnosed by MRI.

**Fig 2:**
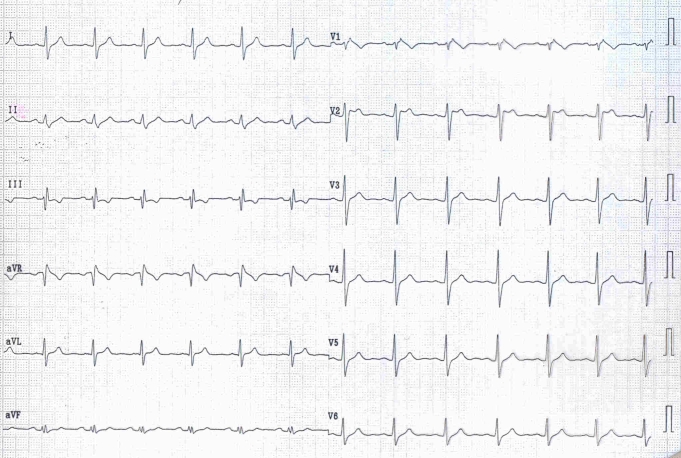
Senior athlete with spontaneous Brugada sign 1 (arrow) on 12–lead ECG.

**Junior athletes.** RSR' (R'height 2.18±0.56mm) recorded in the V1–V3 upper precordial leads, 26 cases (16.56%); questionable Brugada sign in 9 athletes (5.73%). Ajmaline provocative test was negative in 4 cases and refused by 5 subjects. In juniors, the highest incidence of 12–leads ECG abnormalities were J wave elevation (1.32±0.63mm), 34c (27.38%) and ST–segment convex injury elevation (elevation height 2.39±0.79mm, measured at 80ms from J point) pattern, 39c(24.84%) (Table 2). Bifid T waves associated to prolonged QTc intervals (0.56‘± 0.07’) were recorded in 5 subjects (Table 2). Long QTc associated with coved ST elevation in V1 (questionable Brugada sign) in 2 subjects.Fig 2

**Table 2:** 12–lead ECG parameters in senior and junior athletes (SR, sinus rhythm; Arrhyth, arrhythmias; WPW, Wolff– Parkinson–White; Std RSR', standard RSR' in V1–V3 leads; High RSR', higher intercostal spaces; ST– segm, ST–segment elevation myocardial injury like; T>15mm, positive T wave >15mm; * p < 0.01; NS, insignificant)

**12 lead ECG**	**Athletes, seniors N=190 (%)**	**Athletes, juniors N=157 (%)**
**SR**	190 (100)	156 (99.36)
**Arrhyth**	23 (12.10)	19 (12.10)
**WPW**	1 (0.52)	1 (0.63)
**Std RSR' V1–V3 (mm)**	16 (8.42), NS	9 (5.73), NS
**High RSR' V1–V3 (mm)**	45 (23.68) ^*^	26 (16.56) ^*^
**J>0.5 V1–V3 (V4) (mm)**	50 (26.31), NS	43 (27.38), NS
**ST– segm V1–V3 (V4) (mm)**	34 (17.89), NS	39 (24.84), NS
**MT >15 mm V1–V3 (V4)**	23 (12.10), NS	31 (19.74), NS
**Inversed T > 2 mm**	8 (4.21), NS	9 (5.73), NS
**Bifid T wave**	23 (12.10), NS	39 (24.84), NS
**Epsilon wave**	3 (1.57)	–
**Brugada 1 sign**	1 (0.52)	–
**Osborn**	47 (24.73)	37 (23.56)
**QTc int (0.48‘–0.56’)**	8 (4. 21)	7 (4. 45)

Holter method (for 24h) revealed complex premature ventricular beats (n=23 seniors), Wenckebach AV block (n=2 seniors), Wolff–Parkinson–White syndrome (one senior; one junior). Bicuspid aortic valve (n=1), HCM (n=1) and anomalous origin of the RCA from the left Valsalva sinus (n=1) recorded on echocardiography.

### Normal sedentary subjects

According to the decades of age, 505 subjects' ECG characteristics are shown in Table 3. In the first 2 columns, 200 normal subjects (13–20y; 21–30y) match the athletes' age. In this subgroup (13–30 y; n=200) the ECG abnormalities were: RSR’ pattern in the V1–V3 upper precordial leads (n= 24; 12% of 200), J wave elevation (n=13; 6.5% of 200). In the decade of 31–40 years old, one epsilon was recorded and ARVC was diagnosed on  MRI. Typically Brugada 1 sign was detected in 3 cases (decade 21–50y) (Table 3).

N.B. Number, first % (related to age decade group) and second % (related to the 505 controls).

**Table 3:** Electrocardiographic findings in normal sedentary subjects divided by decades of age

**12 lead ECG**	**13–20y, N=21**	**21–30y, N=179**	**31–40y, N=189**	**41–50y, N=67**	**51–60y, N=35**	**61–76y, N=14**
**SR**	21 (100), (4.15)	178 (99.44), (35.24)	189 (100), (37.42)	67 (100), (13.26)	35 (100), (6.93)	14 (100), (2.77)
**WPW**	2 (9.52), (0.39)	3 (1.67), (0.59)	–	2 (2.98), (0.39)	–	–
**Std RSR' V1–V3 (mm)**	1 (4.76), (0.19)	2 (1.17), (0.39)	6 (3.17), (1.18)	3 (4.47), (0.59)	–	–
**High RSR' V1–V3 **	5 (23.8), (0.99)	19 (10.61), (3.76)	24 (12.69), (4.75)	5 (7.46), (0.99)	1 (2.85), (0.19)	–
**J>0.5 V1–V3 (V4) (mV)**	1 (4.76), (0.19)	12 (6.70), (2.37)	18 (9.52), (3.56)	7 (10.44), (1.38)	3 (8.57), (0.59)	–
**ST– segm V1–V3 (V4) (mV)**	–	3 (1.67), (0.59)	–	–	–	–
**T >15 mV, V1–V3 (V4)**	–	2 (1.17), (0.39)	2 (1.05), (0.39)	1 (1.49), (0.19)	–	–
**Inversed T >2 mm**	–	8 (4.47), (1.58)	3 (1.58), (0.59)	1 (1.49), (0.19)	–	1 (7.69), (0.19)
**Epsilon wave**	–	–	1 (0.52), (0.19)	–	–	–
**Brugada 1 sign**	–	1 (0.56), (0.19)	1 (0.52), (0.19)	1 (1.49), (0.19)	–	–
**Osborn**	4 (19.04), (0.79)	33 (18.43), (6.53)	17 (8.99), (3.36)	9 (13.43), (1.78)	2 (5.71), (0.39)	1 (7.69), (0.19)

### Genetic analysis

227 athletes (114 seniors, 113 juniors) and 35 normal sedentary subjects were selected. The 12–lead ECG inclusion criteria for genetic analysis were:

1. Brugada sign;
2. questionable Brugada sign;
3. high RSR‘ precordial leads V1–V3 and R‘ >2mm >R and associated with complex PVB;
4. inverted T waves (>2 mm) in > 2 leads;
5. bifid T wave with distinct two peaks associated or not with prolonged QTc interval;
6. ST–segment convex elevation, injury like pattern;
7. epsilon waves.

**Fig 3:**
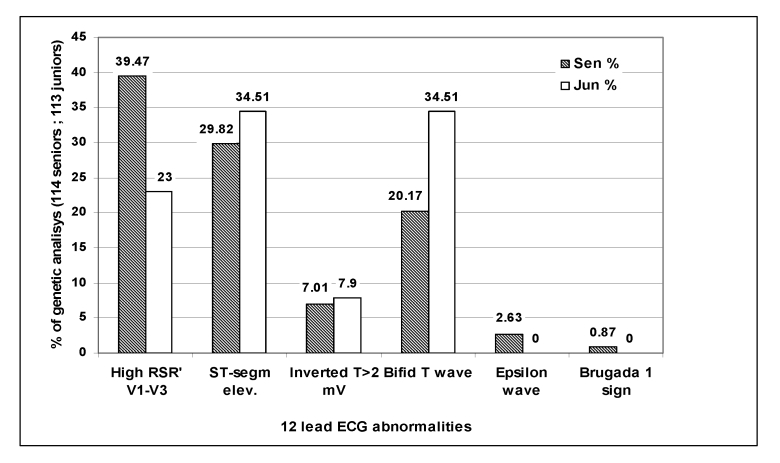
**ECG** abnormalities percent in senior (n=114) and junior (n=113) athletes referred to genetic analysis

In Fig 3 the percent of the ECG abnormalities in senior and junior athletes selected for genetic analyses are presented.

Seven junior athletes had mutations on KCN genes: KCNQ1 (n2), KCNE2 (n1), KCNH2 (n4); all mutations were missense (duplication) on exon 2 (n3), exon 4 (n2), exon 18 (n1) and exon 19 (n1). One of these athletes whose electrocardiograms are presented in Figure 4b,4b' had 2 mutations on 2 different KCN genes (KCNQ1, KCNH2) (Fig 4a).

**Fig 4a:**
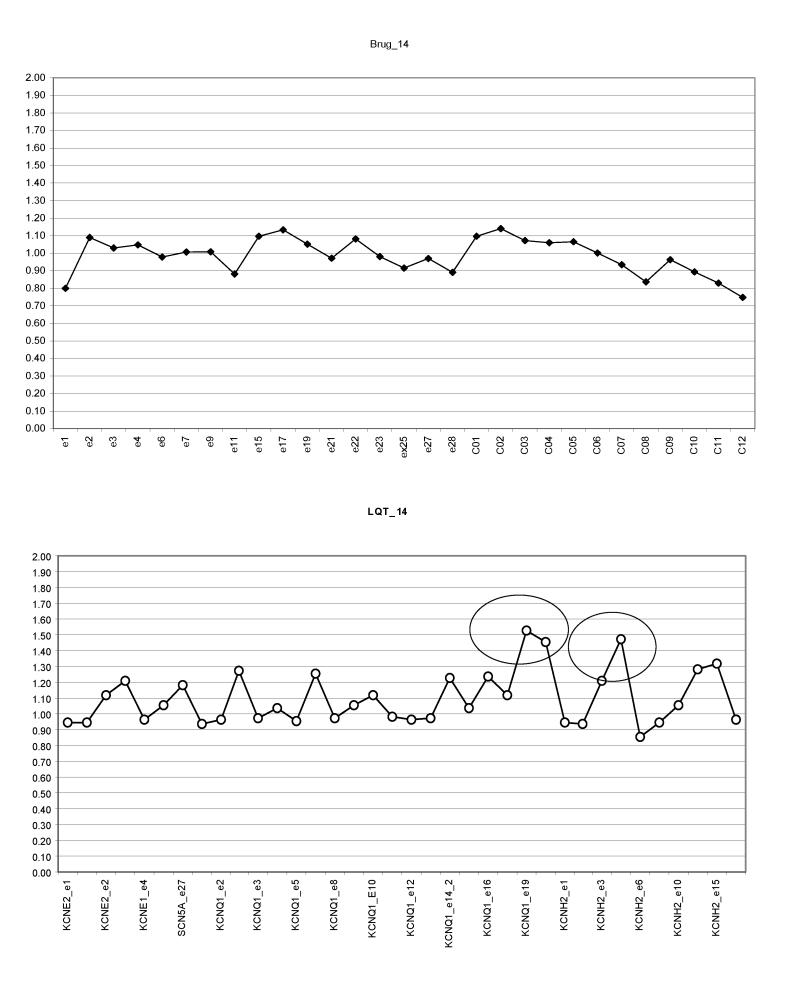
Junior athlete. MLPA profiles for SCN5A gene (SALSA P108, upper panel) and for KCNQ1 and KCNH2 (SALSA P114, lower panel). Possible double mutations (duplication) for exon 18 KCNQ1 and exon 4 KCNH2 (Brug. SALSA P108; LQT. SALSA P114).

**Fig 4b:**
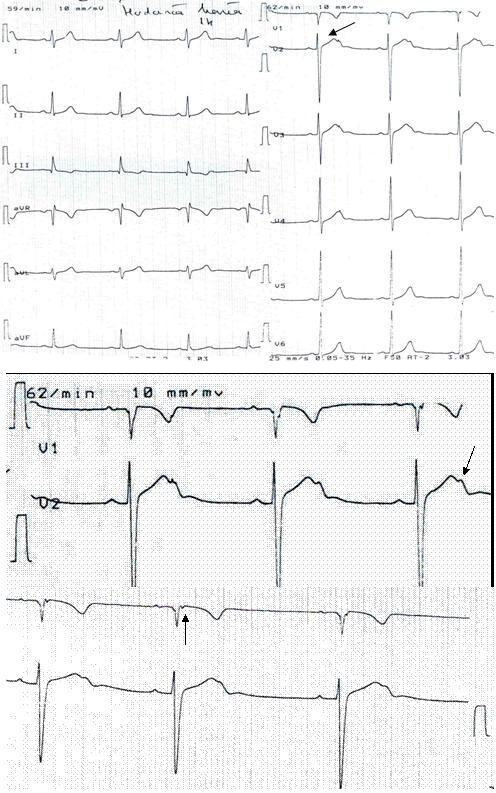
Electrocardiograms of two KCN genes mutations in junior athlete. In standard 12–lead ECG sinus rhythm 62 bpm; bifid T waves and distinct two peaks in V2, V3 precordial leads. (arrow) In higher V1, V2 leads (one upper)–bifid T waves are evident in V2 (arrow); ( two upper spaces) – epsilon wave in V1 (arrow).

**Fig 5:**
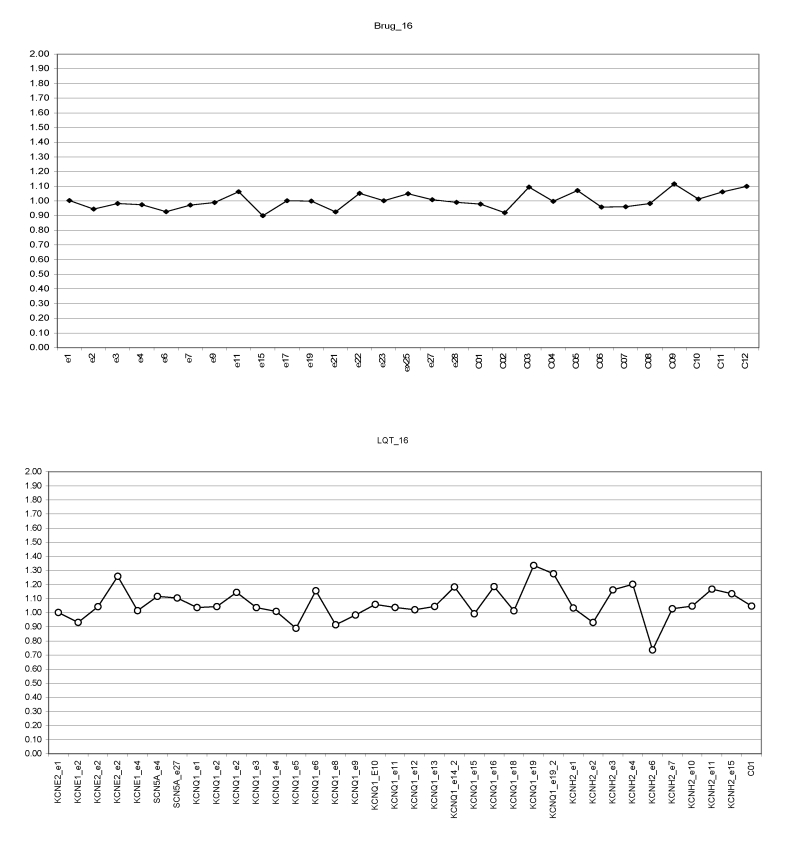
Senior athlete. MLPA profiles for SCN5A gene (SALSA P108, upper panel) and for SCN5A, KCNQ1, KCNH2, KCNE1, KCNE2 (SALSA P114, lower panel). No detectable mutations. (Brug. SALSA P108; LQT. SALSA P114).

Athlete normal genetic profile for SCN5A and KCN genes are shown in Fig 5

**Fig 6:**
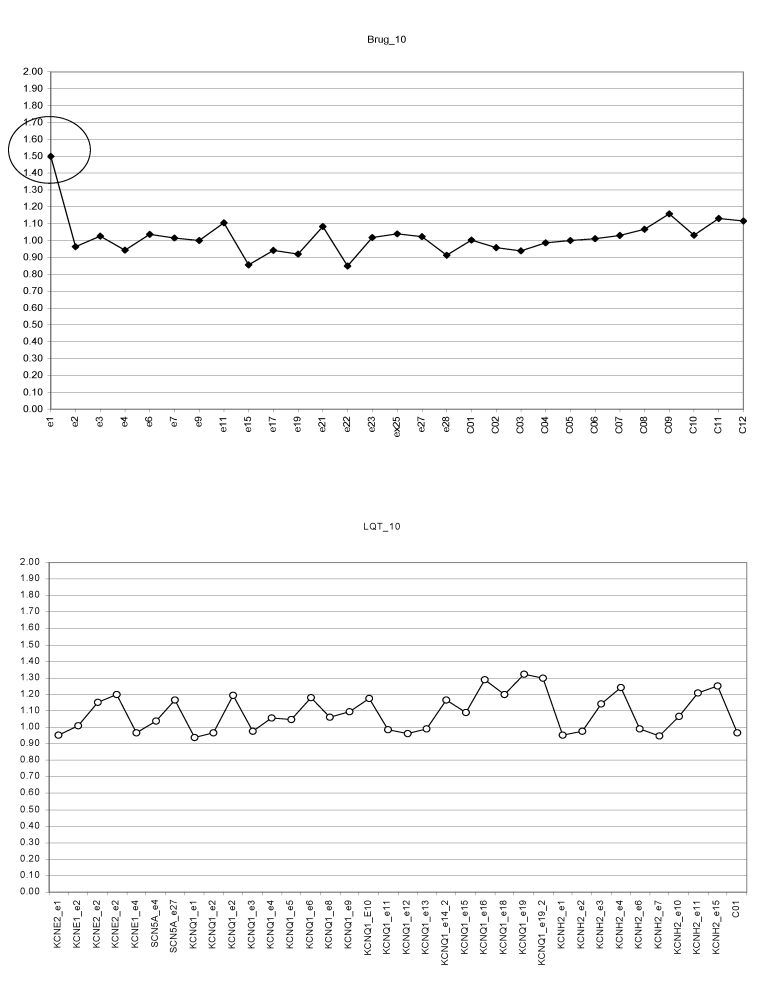
Senior athlete. MLPA profiles for SCN5A (SALSA P108, upper panel) – possible exon1 duplication. SALSA P 114 (lower panel) for KCNE2, KCNE1, KCNQ1, KCNH2 normal profile. Brug. SALSA P108; LQT. SALSA P114).

One senior athlete had missense mutation (duplication) on SCN5A gene, exon1 (Fig 6)

Gene mutations and electrocardiographic associated abnormalities in athletes are summarized in Table 4

N.B. dup, duplication; e, exon

**Table 4:** CGenes mutations and ECG abnormalities in 7 athletes

**Athletes**	**KCNQ1**	**KCNE2**	**KCNH2**	**SCN5A**	**ECG**
**1**	dup e 18	–	–	–	bifid T wave; QTc 0.52
**2**	–	dup e 2	–	–	bifid T wave; QTc 0.56
**3**	dup e 19 (19–1;19–2)	–	dup e 4	–	bifid T wave; QTc 0.48–0.50
**4**	–	–	dup e 4	–	QTc 0.56
**5**	–	–	dup e 2	–	inverted T wave; QTc 0.48
**6**	–	–	dup e 2	–	questionable Brugada
**7**	–	–	–	dup e 1	questionable Brugada

In sedentary normal subjects, one subject with Brugada 1 sign and his healthy and electrocardiographically normal mother had similar mutations (deletion) on SCN5A gene, exon1. An epsilon sedentary normal subject (decade 31– 40y) had MRI documented ARVC; no gene mutations were detected, but his clinically and electrocardiographically normal sister had missense mutation (deletion) on SCN5A, exon1.

## Discussions

Since 2004 we have been interested to evaluate the ECG patterns in highly trained athletes from different level of training (juniors, seniors) involved in sports with intensive cardiovascular burden. We observed ECG tracings with bizarre and unexpected QRS and ST–T aspects, different from the ‘classically’ ECG patterns described in elite endurance athletes [20, 58–61].

The main purpose of our study was to find out whether these ECG (V1–V3) morphologies (possible delayed impulse conduction in the right ventricle, including RVOT) were linked to genetic mutations responsible for inherited channelopathies (i.e. Brugada syndrome, RVOT ventricular tachycardias) and cardiomyopathies (i.e.ARVC). Gene mutations for HCM in subjects with specific ECG abnormalities were searched for.

The senior and junior athletes' electrocardiograms were analyzed separately and compared to sedentary normal, aged matched persons' ECG data. In seniors, the RSR' pattern dominate with R'>2mm in higher V1–V3 leads; 34% of RSR' athletes had ST–segment elevation, resembling the Brugada sign present in only one lead and V1 (questionable Brugada sign) in few cases. Possible abnormal (delayed) impulse conduction into RVOT has been considered regarding this mechanism [49].

The highest incidence of junior ECG abnormalities was elevated J wave and convex injury like ST–segment elevation in V1–V3,V4 leads (standard, high). In junior athletes, bifid T wave associated to moderately long QTc interval arose the question of a possible abnormal prolonged ventricular repolarization. Few of our controls had similar ECG abnormalities.

A molecular substratum not evident in ordinary life but exacerbated by strenuous training could be the cause of athletes' ECG abnormalities.

To support this hypothesis we focused on the relation between the ECG patterns suggestive of Brugada syndrome, long QT syndrome, HCM, ARVC and specific genetic study.

The genetic analysis referred to mutations in SCN5A and LQTS genes and genes responsible for inherited cardiomyopathies.

Junior athletes had 7 mutations in LQTS genes (KCNQ1, KCNH2, KCNE2) one of them with two KCN mutations, on KCNQ1 and KCNH2. All seven abnormalities were missense (duplication) mutations on exons 2 ,4, 18 and 19. 

Three controls had SCN5A mutations: two of these (deletions) were in 1 Brugada person and his healthy mother. Interestingly, a family member (healthy sister) of a documented ARVC patient had a SNC5A mutation (duplication). All SCN5A mutations were missense, heterozygote type.

No specific molecular abnormalities were found in other athletes.

The mutations identified by MLPA will be detailed by complex genetic analyses such as the sequence techniques.

## Conclusions

The electrocardiogram in athletes performing high endurance training is sometimes strange, looking like inherited channelopathy and cardiomyopathy ECG patterns. Specific tests for correct diagnosis are frequently necessary. Genetic analysis, the ‘last step’ to diagnosis sometimes gives surprising and unexpected information, whose significance needs to be investigated. Complex molecular techniques could bring forward details of these genetic mutations identified in elite athletes with ‘frequently encountered 12–lead ECG athletics patterns’.

The next and future medical approach regarding the athletic performance is still a challenge for the scientific medical community.

## Acknowledgement

This study was supported by a grant from the Ministry of Education and Research, Romania

‘Genomics and Proteomics Project’

**Grant**: The genetic mutations screening in inherited arrhythmic syndromes. Genotype–phenotype correlations from the clinical management point of view.

Abbreviations:

- EP– electrophysiologic study
- ECG = electrocardiographic/electrocardiography
- HCM=hypertrophic cardiomyopathy
- ARVC = arrhythmogenic right ventricular cardiomyopathy
- RVOT=right ventricular outflow tract
- RCA=right coronary artery
- PVB=premature ventricular beats
